# Mechanisms of Breast Cancer Stem Cell Specification and Self-Renewal Mediated by Hypoxia-Inducible Factor 1

**DOI:** 10.1093/stcltm/szad061

**Published:** 2023-09-28

**Authors:** Gregg L Semenza

**Affiliations:** Armstrong Oxygen Biology Research Center, Institute for Cell Engineering, and Department of Genetic Medicine, Johns Hopkins University School of Medicine, Baltimore, MD, USA

**Keywords:** breast cancer stem cells, hypoxia, pluripotency factors, self-renewal, telomerase

## Abstract

Many advanced human cancers contain regions of intratumoral hypoxia, with O_2_ gradients extending to anoxia. Hypoxia-inducible factors (HIFs) are activated in hypoxic cancer cells and drive metabolic reprogramming, vascularization, invasion, and metastasis. Hypoxia induces breast cancer stem cell (BCSC) specification by inducing the expression and/or activity of the pluripotency factors KLF4, NANOG, OCT4, and SOX2. Recent studies have identified HIF-1-dependent expression of PLXNB3, NARF, and TERT in hypoxic breast cancer cells. PLXNB3 binds to and activates the MET receptor tyrosine kinase, leading to activation of the SRC non-receptor tyrosine kinase and subsequently focal adhesion kinase, which promotes cancer cell migration and invasion. PLXNB3-MET-SRC signaling also activates STAT3, a transcription factor that mediates increased *NANOG* gene expression. Hypoxia-induced NARF binds to OCT4 and serves as a coactivator by stabilizing OCT4 binding to the *KLF4*, *NANOG,* and *SOX2* genes and by stabilizing the interaction of OCT4 with KDM6A, a histone demethylase that erases repressive trimethylation of histone H3 at lysine 27, thereby increasing *KLF4*, *NANOG,* and *SOX2* gene expression. In addition to increasing pluripotency factor expression by these mechanisms, HIF-1 directly activates expression of the *TERT* gene encoding telomerase, the enzyme required for maintenance of telomeres, which is required for the unlimited self-renewal of BCSCs. HIF-1 binds to the *TERT* gene and recruits NANOG, which serves as a coactivator by promoting the subsequent recruitment of USP9X, a deubiquitinase that inhibits HIF-1α degradation, and p300, a histone acetyltransferase that mediates acetylation of H3K27, which is required for transcriptional activation.

**Significance Statement**

Cancer stem cells are a small subpopulation of cells that are capable of tumor initiation and infinite self-renewal. Intratumoral hypoxia induces HIF-1, which activates *PLXNB3, NARF*, and *TERT* gene expression, leading to increased expression and activity of pluripotency factors and telomerase, which mediate breast cancer stem cell specification and self-renewal, respectively. Development of HIF-1 inhibitors may provide a novel therapeutic strategy for the eradication of breast cancer stem cells.

## Intratumoral Hypoxia

The pioneering work of Peter Vaupel and his colleagues established that one of the unifying features of advanced human cancers is the presence of intratumoral hypoxia. Using Eppendorf microelectrodes, pO_2_ was directly measured in accessible tumors, such as those of the breast, head/neck, and uterine cervix. Based on 1009 measurements from 16 subjects, Vaupel’s group found a broad range of pO_2_ values in normal breast tissue from 15 to 100 mmHg, with median pO_2_ = 65 mmHg; by contrast, in 851 measurements from 15 breast cancers, the median pO_2_ was 10 mmHg, with the most common measurement values = 0-5 mmHg.^1^ This profound intratumoral hypoxia reflects a severe mismatch between O_2_ supply and demand, with supply impaired by the presence of blood vessels that are structurally and functionally abnormal, and demand driven by dysregulated cell proliferation.^1,2^ In the uterine cervix, a large difference in pO_2_ between normal and cancer tissue (42 and 9 mmHg, respectively) was also reported and pO_2_ < 10 mmHg in cervical cancer was associated with decreased disease-free and overall survival.^3^

Within each tumor, pO_2_ levels vary according to the distance from the nearest functional blood vessel. O_2_ undergoes radial diffusion from blood vessels and is consumed by cells for respiration. In many tumors, adequate O_2_ is available to cancer cells that are less than 100 µm from the nearest blood vessel and these cells are said to be normoxic, whereas when cancer cells are located greater than 100 µm from a vessel, O_2_ becomes limiting and the cells are said to be hypoxic; and, in many tumors, when cells are located greater than 200 µm, O_2_ has become depleted and the cells are said to be anoxic (and dead). In their landmark 1955 paper, Thomlinson and Gray concluded that O_2_ depletion was the primary factor leading to tumor necrosis.^4^

## Hypoxia-Inducible Factors

Decreased O_2_ availability triggers the accumulation of hypoxia-inducible factors (HIFs). HIFs are heterodimeric proteins that consist of an O_2_-regulated HIF-α subunit (HIF-1α, HIF-2α, or HIF-3α)and a constitutively expressed HIF-1β subunit, as originally determined for HIF-1.^5,6^ Under normoxic conditions, the HIF-α subunits are subjected to hydroxylation on 2 proline residues (P402 and P564 in human HIF-1α), leading to binding of the von-Hippel-Lindau (VHL) protein, which targets HIF-α proteins for ubiquitination and proteasomal degradation; under hypoxic conditions, the hydroxylation reaction (which uses O_2_ as a substrate) is inhibited, and the non-hydroxylated HIF-α subunits rapidly accumulate, dimerize with HIF-1β, bind to the consensus sequence 5ʹ-RCGTG-3ʹ (R = A or G) at target genes and activate their transcription.^7-9^

Immunohistochemistry using antibodies against HIF-1α to analyze primary tumor biopsies has demonstrated that increased HIF-1α expression is associated with increased patient mortality in bladder, brain, breast, cervical, colorectal, endometrial, esophageal, gastric, head/neck/ oropharyngeal, hepatocellular, lung, ovarian, and pancreatic cancer as well as acute leukemias and melanoma.^9,10^ In many cancers, HIF-1α expression is observed in a peri-necrotic pattern, representing the viable cells that are furthest away from a blood vessel and therefore the most hypoxic. However, HIF-1α can also be activated by oncogenic mutations in an O_2_-independent manner (most notably in the clear cell type of renal cell carcinoma which is characterized by VHL loss-of-function), leading to HIF-1α expression throughout the tumor biopsy.^11^ Focal, hypoxia-induced vs diffuse, O_2_-independent HIF-1α expression patterns were observed in two-thirds and one-third of oropharyngeal cancers, respectively.^12^ Exposure of SUM159 human breast cancer cells (see Table 1 for characterization of breast cancer cell lines mentioned in this review) to hypoxia (1% O_2_ for 24 h) led to increased expression of hundreds of RNAs and decreased expression of hundreds of RNAs in a HIF-dependent manner.^13^ Similar results have been observed in every other type of cancer, with each cancer cell line showing a unique pattern of altered gene expression in response to hypoxia.^14^ These HIF-dependent changes in gene expression promote vascularization, metabolic reprogramming, migration, invasion, metastasis, and immune evasion.^7,8,10,15-17^

**Table 1.** Expression of estrogen receptor (ER), progesterone receptor (PR), human epidermal growth factor receptor 2 (HER2) and BRCA1 tumor suppressor protein in breast cancer cell lines described in this review.

Cell line	Species	ER	PR	HER2	BRCA1
4T1	Mouse	−	−	−	+
HCC1954	Human	−	−	+	+
MCF−7	Human	+	+	-	+
MDA-MB-231	Human	−	−	−	+
SUM149	Human	−	−	−	−
SUM159	Human	−	−	−	+
T47D	Human	+	+	−	+

## Breast Cancer Stem Cells

Cancer stem cells (CSCs), which were first discovered in acute myeloid leukemia,^18,19^ have been identified in many types of cancer, including brain tumors^20^; breast,^21^ colon,^22^ head/neck,^23^ pancreatic,^24^ and prostate^25^ cancer; melanoma^26^; and neuroblastoma.^27^ CSCs are defined by their ability to self-renew by asymmetrically dividing into one CSC, which is capable of infinite cell divisions, and one transient amplifying cell, which is capable of rapid cell division but only for a finite number of divisions.^28^ CSCs are the only cells within a tumor that are capable of giving rise to a secondary (recurrent or metastatic) tumor.^29-31^ CSCs are specified and maintained by the expression of core pluripotency factors, which were originally identified in embryonic stem cells (ESCs), including octamer binding factor 4 (OCT4), SRY-box 2 (SOX2), Kruppel-like factor 4 (KLF4), and NANOG.^32-35^ Cell populations that are enriched for breast CSCs (BCSCs) can be identified by flow cytometry as CD44^hi^CD24^lo,21^ except in many triple-negative breast cancer (TNBC) cell lines, which constitutively express CD44.^36^

BCSCs express aldehyde dehydrogenase 1 (ALDH1), which oxidizes retinaldehyde to retinoic acid, which induces gene expression by binding to retinoic acid receptors (RARs) and licensing their transcriptional activity.^37^ ALDH1 is directly linked to the pluripotency factors, as KLF4 and SOX2 have been reported to activate expression of one or more ALDH1 genes (*ALDH1A1, ALDH1A2, ALDH1A3*) in breast cancer.^38,39^ High ALDH1 expression (as measured by ALDH enzyme activity detected by use of a fluorogenic substrate and flow cytometry) is correlated with increased risk of metastasis and patient mortality.^37,40,41^ BCSCs propagate as multicellular spheroids (mammospheres) when placed in suspension culture using ultra-non-adherent plates.^42^ The primary mammospheres can be harvested, dissociated into single cells, and replated to assay for secondary spheroid formation as a measure of cell renewal.^43^ In addition to self-renewal and limitless cell division, BCSCs have tumor-initiating properties: 500 ALDH1^+^ human breast cancer cells will reliably form tumors when injected into the mammary fat pad of immunodeficient mice, whereas 50 000 ALDH1^−^ cells from the same breast cancer will not.^37^ Selection of CD44^hi^CD24^lo^ cells from human breast cancers also enriches for tumor-initiating cells.^21^

## Hypoxia, HIF-1, and BCSCs

Exposure of human breast cancer cells to 1% O_2_ for 3 days increases the percentage of BCSCs by several fold and this increase in BCSC specification is dependent on the induction of multiple HIF-1 target genes but is independent of HIF-2.^44,45^ In contrast, the enrichment of BCSCs among cells surviving chemotherapy is dependent on both HIF-1 and HIF-2.^45^ In 4T1 mouse mammary carcinoma cells, HIF-2α expression was found to be enriched among ALDH1^+^ cells, treatment with an ALDH1 inhibitor led to decreased HIF-2α mRNA expression, and HIF-2α knockdown by RNA interference led to decreased OCT4 mRNA expression in these cells under non-hypoxic conditions.^46^ Knockdown of ALDH1A1 expression in MCF-7 cells impaired mammosphere formation, whereas ALDH1A1 overexpression increased mammosphere formation and increased the expression of the stemness markers CD133 and KLF4.^47^ Furthermore, changes in ALDH1A1 expression were associated with corresponding changes in the expression of HIF-1α and VEGFA, which is the product of a HIF-1 target gene.^48^ Remarkably, exposure of ALDH1A1-knockdown cells to exogenous retinoic acid restored HIF-1α and VEGFA expression, whereas exposure of cells overexpressing ALDH1A1 to the RAR inhibitor AGN-193109 led to decreased HIF-1α and VEGFA expression.^47^ HIF-1α knockdown inhibited expression of both VEGFA and CD133, thereby placing HIF-1 both upstream and downstream of stemness markers in MCF-7 cells. ALDH1A1 → HIF-1α → VEGFA signaling triggered angiogenesis in MCF-7 tumor xenografts.^47^ This result, like many others, demonstrates that BCSC specification is simply a component (along with angiogenesis, migration/invasion, metabolic reprogramming, and chemotherapy resistance) of the “high HIF” phenotype, also known as the lethal cancer phenotype.

**Figure 1:**
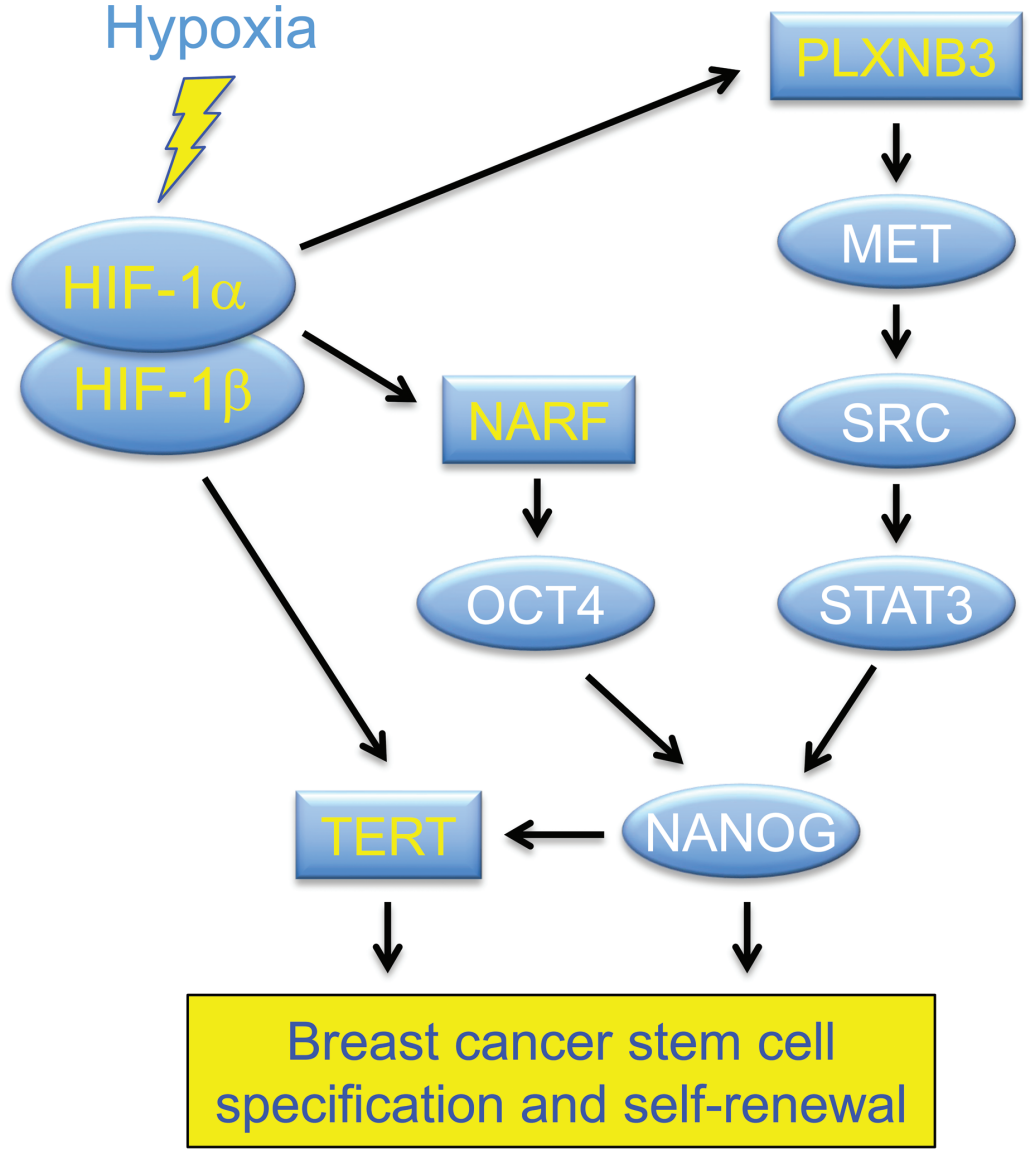
Hypoxia induces HIF-1-dependent transcriptional activation of the *PLXNB3, NARF,* and *TERT* genes, leading to increased breast cancer stem cell specification and self-renewal.

In contrast to BCSCs, exposure of human ESCs to hypoxia and reoxygenation was shown to induce the expression of pluripotency factors in a HIF-2-dependent manner^49^ and hypoxia induced the binding of HIF-2 to the promoters of the genes encoding NANOG, OCT4, and SOX2.^50^ Hypoxia also induced pluripotency factor expression in many cancer cell lines in a HIF-dependent manner.^51^ In hypoxic breast cancer cells, the mechanism of induction appears to be indirect: multiple HIF-1 target genes encode proteins that activate signal transduction pathways leading to increased expression or activity of NANOG, OCT4, and other pluripotency factors (Fig. 1). This principle is illustrated by several recent studies that are described below, which have implicated PLXNB3 and NARF as novel mediators of breast cancer stem cell specification and elucidated a novel mechanism by which telomerase activity is reactivated in breast cancer stem cells.

### PLXNB3

Plexin-B3 (PLXNB3) is a large transmembrane protein that interacts with semaphorin 5A to mediate axon guidance during the development of the nervous system.^52^ PLXNB3 mRNA is expressed in many cancers and is associated with increased pan-cancer patient mortality.^53^ However, PLXNB3 was not previously implicated in cancer stem cell specification. PLXNB3 mRNA expression was induced by hypoxia in SUM159 human TNBC cells as part of a battery of RNAs associated with axon guidance^13^ and was correlated with the expression of a HIF metagene signature in 1218 human breast cancers in The Cancer Genome Atlas (TCGA BRCA dataset).^54^ Immunohistochemical analysis revealed that HIF-1α and PLXNB3 protein expression were highly correlated in human breast cancers and that PLXNB3 protein expression greater than the median was associated with decreased overall survival of women with breast cancer.^54^

**Figure 2:**
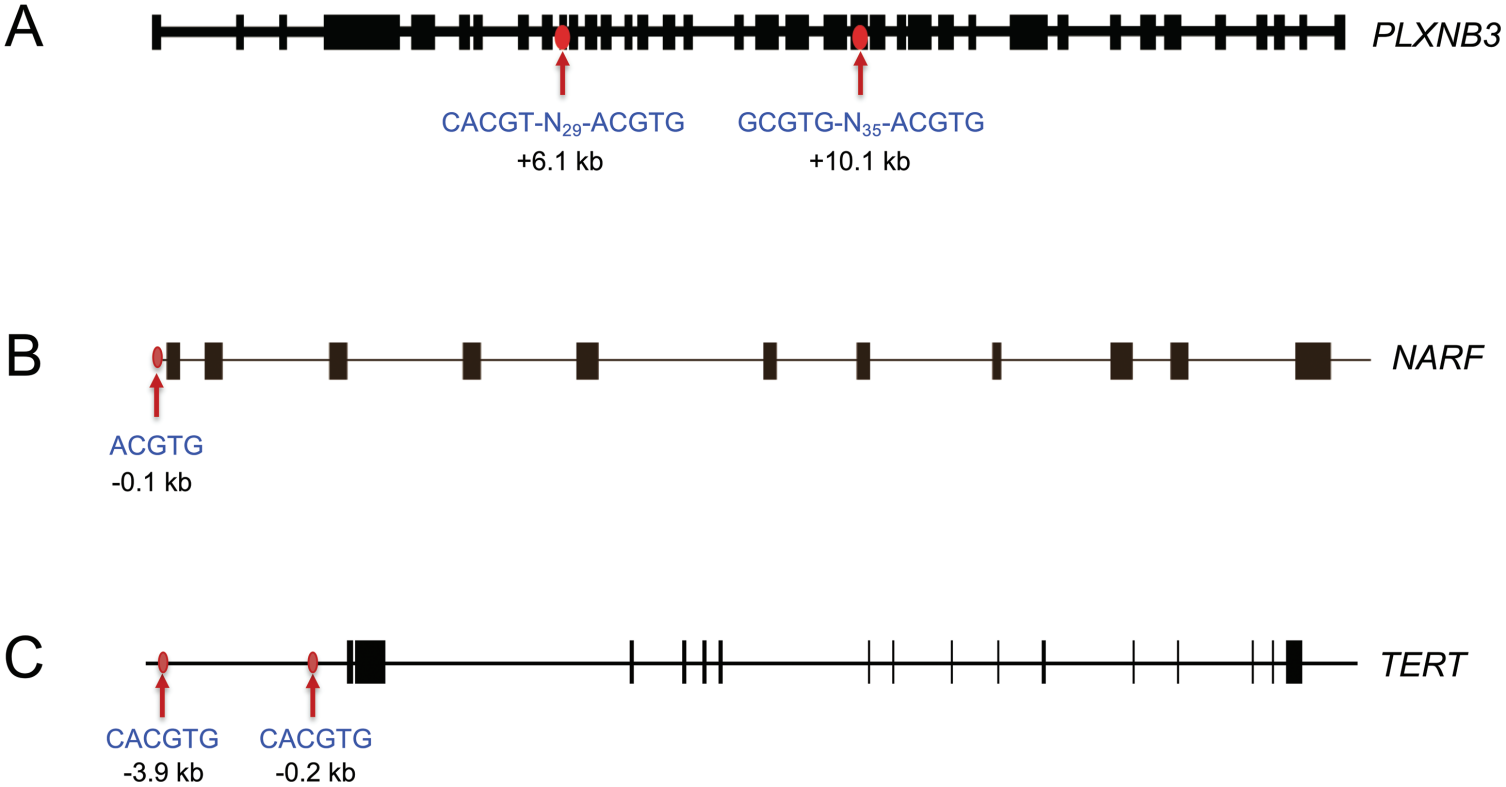
Localization of HIF-1 binding sites in the *PLXNB3*^51^ (**A**)*, NARF*^55^ (**B**), and *TERT*^56^ (**C**) genes. Chromatin immunoprecipitation identified hypoxia-induced binding of HIF-1α and HIF-1β to genomic regions containing sequences (shown beneath the arrows) that match the HIF consensus binding site 5ʹ-RCGTG-3ʹ (R = A or G) or its complement 5ʹ-CACGY-3ʹ (Y = C or T). Note that these genes contain from 1 to 4 HIF consensus sequences in various orientations at locations (denoted by arrows) which are either upstream or downstream of the transcription start site (at the 5ʹ end of the first exon). Exon-intron structures of the genes are shown extending from left to right in the same orientation as the sequences.

Expression of short hairpin RNA (shRNA) targeting HIF-1α in triple-negative SUM159 or human epidermal growth factor receptor 2-positive (HER2^+^) HCC1954 breast cancer cells blocked the hypoxic induction of PLXNB3 mRNA and protein, whereas expression of shRNA targeting HIF-2α had no effect. Chromatin immunoprecipitation (ChIP) assays revealed the hypoxia-induced binding of HIF-1α and HIF-1β, but not HIF-2α, at 2 sites located approximately 6 and 10 kb downstream of the transcription start site of the *PLXNB3* gene in SUM159 and HCC1954 cells (Fig. 2A), demonstrating that *PLXNB3* is direct HIF-1 target gene.^54^

Exposure of HCC1954 and SUM159 cells to hypoxia induced: increased migration and invasion; increased ALDH1 and NANOG expression; and enrichment of CD24^−^CD44^+^ and mammosphere-forming cells, all of which were lost in subclones expressing shRNA targeting PLXNB3.^54^ Since migration, invasion, and CSC properties are all fundamental to metastatic breast cancer cells, we injected MDA-MB-231 subclones with altered PLXNB3 expression into the mammary fat pad of immunodeficient female mice. The parental MDA-MB-231 TNBC cells form primary tumors that spontaneously metastasize to the lungs. Primary tumor growth was not significantly affected by the expression of either of the 2 different shRNAs targeting PLXNB3. However, metastasis to the lungs was virtually eliminated in the absence of PLXNB3 expression. When limiting numbers of MDA-MB-231 cells were injected into the mammary fat pad (1 × 10^3^ rather than 2 × 10^6^ used in the prior experiment), tumors formed in 10 out of 10 mice injected with cells expressing a non-targeting control (NTC) shRNA but in only 3 or 4 of out of 10 mice injected with cells expressing either of 2 different shRNAs targeting PLXNB3, demonstrating a significant impairment of tumor formation by PLXNB3 loss of function.^54^

The studies described above provide molecular, cellular, and in vivo data indicating the PLXNB3 is essential for BCSC specification. But what is the mechanism? First, in SUM159 cells, the knockdown of SEMA5A, which is the specific ligand for PLXNB3,^52^ impaired hypoxia-induced migration, invasion, and enrichment of ALDH1^+^ and mammosphere-forming cells, similar to the effect of PLXNB3 knockdown.^54^ Second, activation of PLXNB3 by SEMA5A is known to enable PLXNB3 to interact with, and activate, the MET receptor tyrosine kinase, even in the absence of hepatocyte growth factor, which is the cognate ligand for MET.^52^ In many cancers, MET signals to the non-receptor tyrosine kinase SRC,^57^ which activates focal adhesion kinase (FAK), which in turn is essential for BCSC specification (Fig. 1) as well as breast cancer invasion and metastasis.^55,58,59^ In SUM159 cells, both hypoxia-induced invasion of Matrigel (a tumor basement membrane preparation) and enrichment of ALDH1^+^ BCSCs was blocked by treatment with an inhibitor of MET, SRC, or FAK.^54^ SRC also signaled to STAT3, which activates *NANOG* transcription, and hypoxia-induced NANOG mRNA and protein expression were blocked by treatment with an inhibitor of MET, SRC, or STAT3.^54^ Thus, oncogenic signal transduction pathways leading to BCSC specification and metastasis are activated, in the absence of genetic alterations, within the hypoxic tumor microenvironment.

### NARF

RNA-seq data from SUM159 cells revealed that hypoxia-induced the expression of NARF mRNA,^13^ which encodes nuclear prelamin A recognition factor, a poorly characterized nuclear protein that is also known as iron-only hydrogenase-like protein 2 (despite its name, it has no hydrogenase activity).^60,61^ NARF has not been previously implicated in breast cancer pathogenesis or cancer stem cell biology. The reader should be aware that the acronym NARF has also been applied to the nemo-like kinase ring finger protein, which is the product of the *RNF138* gene. NARF expression was induced by hypoxia in all breast cancer cell lines analyzed, including hormone (estrogen and/or progesterone) receptor-positive (HR^+^) MCF-7 and T47D cells, HER2^+^ HCC1954 cells, and triple-negative (HR^−^ and HER2^−^) MDA-MB-231, SUM149, and SUM159 cells.^62^ Knockdown experiments in HR^+^ MCF-7 and HR^−^ MDA-MB-231 cells revealed that hypoxia-induced NARF expression was dependent on HIF-1α and independent of HIF-2α. This conclusion was supported by ChIP assays demonstrating hypoxia-induced binding of HIF-1α and HIF-1β, but not HIF-2α, at a consensus HIF binding site (5ʹ-ACGTG-3ʹ) located 95 bp 5ʹ to the transcription start site of the *NARF* gene (Fig. 2B). A 55-bp sequence encompassing the site functioned as a hypoxia response element when inserted into a luciferase reporter plasmid, whereas a 3-bp mutation in the HIF binding site (ACGTG to AAAAG) eliminated hypoxia-induced luciferase expression.^62^ These studies demonstrated that *NARF* was a direct HIF-1 target gene.

In 1218 breast cancers (TCGA BRCA dataset), NARF mRNA expression was significantly correlated with a 10-gene HIF signature (*R* = 0.42; *P* < .0001) and a 20-gene BCSC signature (*R* = 0.50; *P* < .0001). Hypoxia-induced enrichment of ALDH1^+^ cells and mammosphere-forming cells was impaired in MDA-MB-231 and MCF-7 subclones with NARF knockdown.^62^ When MDA-MB-231 subclones were injected into the mammary fat pad of immunodeficient mice (2 × 10^6^ cells, such that BCSCs were not limiting for tumor initiation), there was no difference in primary tumor growth rate between cells expressing a non-targeting control shRNA (NTC) and cells expressing either of 2 different shRNAs targeting NARF, but lung metastasis of NARF-knockdown cells was markedly impaired. When only 1000 cells were injected (such that BCSCs were limiting), tumors formed in 10/10 mice injected with NTC cells, but only 3/10 and 2/9 mice injected with either of 2 NARF-knockdown subclones, indicating that NARF plays a critical role in BCSC specification and tumor initiation, as described above for PLXNB3. Analysis of human breast cancer biopsy tissues by immunohistochemistry revealed that high NARF protein expression was associated with decreased overall survival of breast cancer patients.^62^

Exposure of HR^+^ MCF-7 and HR^−^ MDA-MB-231 cells to hypoxia induced the NARF-dependent expression of KLF4, NANOG, and SOX2 mRNA and protein, whereas OCT4 (encoded by the *POU5F1* gene) was constitutively expressed and was not affected by NARF knockdown.^62^ In MDA-MB-231 cells, OCT4 bound to, and was required for expression of, the *KLF4*, *NANOG,* and *SOX2* genes. NARF was also required for hypoxia-induced expression of KLF4, NANOG, and SOX2.^62^ NARF was recruited to OCT4 binding sites in the *KLF4*, *NANOG,* and *SOX2* genes in an OCT4-dependent manner, and NARF, in turn, increased the occupancy of OCT4 at these sites. In contrast, OCT4 binding to the *POU5F1* gene was neither hypoxia-induced nor NARF-dependent. Hypoxia-induced recruitment of the histone-lysine demethylase KDM6A by NARF and OCT4 to the *KLF4*, *NANOG,* and *SOX2* genes led to decreased trimethylation of lysine-27 of histone H3 (H3K27me3, a repressive histone mark) and increased gene expression.^62^ In contrast, KDM6A recruitment to the OCT4 site in the *POU5F1* gene was neither hypoxia-induced nor NARF-dependent. These data indicate that whereas OCT4 autoregulates its own expression in a constitutive manner, the ability of OCT4 to increase the expression of KLF4, NANOG, and SOX2 in response to hypoxia is dependent on the induction of NARF and its association with both OCT4 and KDM6A. Thus, NARF functions as a coactivator for OCT4, stabilizing its binding to DNA and promoting the recruitment of KDM6A, which erases the repressive histone mark H3K27me3, thereby facilitating transcription of the *KLF4*, *NANOG,* and *SOX2* genes.

### TERT

While the ability of pluripotency factors such as NANOG and OCT4 to regulate the expression of one another is well established, the targets of these transcription factors that mediate self-renewal are less well studied. Telomeres are several-kilobase-long repeats of the sequence 5ʹ-TTAGGG-3ʹ at the tips of each human chromosome.^63^ The maintenance of telomere length by the enzyme telomerase is required for the infinite self-renewal of ESCs and CSCs.^28,64^ Telomerase consists of the telomerase reverse transcriptase (TERT) and an RNA that serves as a template for reverse transcription.^65^ TERT is expressed during embryogenesis, repressed in adult cells, and reactivated in BCSCs, although *TERT* gene amplification and promoter mutations are rare in common forms of breast cancer, thereby leaving the mechanism of reactivation unexplained.^66-68^ Hypoxia-induced TERT expression mediated by HIF-1 has been reported in several types of cancer^56,69,70^ as well as ESCs,^71^ but the mechanism that restricts HIF-1-dependent TERT expression to stem cells has not been determined.

NANOG knockdown in MDA-MB-231 cells significantly decreased the percentage of ALDH1^+^ cells and mammosphere-forming cells under both hypoxic and non-hypoxic culture conditions.^72^ NANOG overexpression increased the expression of KLF4, OCT4, and SOX2 but did not increase the percentage of ALDH1^+^ cells or mammosphere-forming cells under non-hypoxic conditions. In contrast, NANOG overexpression did increase the percentage of BCSCs under hypoxic culture conditions and this effect was lost in HIF-1α-knockdown cells. Overexpression of NANOG with HIF-1α, but not with HIF-2α, increased the percentage of BCSCs under non-hypoxic conditions.^72^ These results indicated that NANOG is necessary but not sufficient for BCSC specification and suggested that both NANOG and HIF-1α were required for BCSC specification.

TERT knockdown in MDA-MB-231 cells resulted in progressive telomere shortening and loss of proliferative ability after 6 passages.^72^ The percentage of ALDH1^+^ cells also decreased progressively as TERT-knockdown subclones were passaged. Exposure of ER^+^ MCF-7 and ER^-^ MDA-MB-231 cells to hypoxia increased TERT mRNA and protein expression, telomerase activity, and telomere length, all of which were lost by knockdown of HIF-1α but not HIF-2α.^72^ ChIP assays identified hypoxia-induced binding of HIF-1α and HIF-1β at 2 sites located approximately 0.2 and 3.9 kb 5ʹ to the *TERT* gene (Fig. 2C). TCGA BRCA dataset analysis revealed that the 10-gene HIF signature was significantly correlated with a 43-gene telomerase signature,^73^ suggesting that HIF-1 plays a key role in regulating telomerase activity in primary human breast cancers.^72^

NANOG knockdown blocked hypoxia-induced TERT expression, similar to the effect of HIF-1α knockdown. NANOG knockdown also blocked hypoxia-induced telomerase activity and telomere lengthening, similar to the effect of HIF-1α or TERT knockdown. ChIP assays revealed that NANOG was recruited to HIF-1 binding sites in the *TERT* gene and that NANOG knockdown impaired the hypoxia-induced binding of HIF-1α and HIF-1β to the TERT promoter. NANOG interacted directly with the transactivation domain of HIF-1α and recruited USP9X, a deubiquitinase, to HIF-1α, thereby blocking its ubiquitination and proteasomal degradation. In addition, NANOG interacted with the histone acetyltransferase p300 to stabilize its interaction with HIF-1α, thereby increasing acetylation of histone H3 at lysine residue 9 (H3K9ac) and H3K27ac, which are histone marks associated with transcriptional activation, at the *TERT* promoter.^72^ Thus, NANOG functions as a coactivator that increases HIF-1α protein stability and transactivation function in breast cancer cells.

## Conclusions

BCSCs are the only cells within a primary tumor capable of giving rise to a secondary (recurrent and/or metastatic) tumor, based on their ability for infinite self-renewal, which is controlled by the expression of pluripotency factors that were first described in ESCs. However, unlike ESCs, BCSCs are not pluripotent and rather than representing a developmental state, they are better characterized as representing a reversible physiological state that is induced by intratumoral hypoxia. Indeed, the studies described above indicate that an intimate, cooperative relationship between HIF-1 and NANOG is required for BCSC specification and for self-renewal, which is dependent upon TERT expression. Moreover, BCSCs are inherently invasive and metastatic, as demonstrated in the PLXNB3 study. HIFs also activate the expression of genes encoding proteins that mediate immune evasion by cancer cells.^15,17^ Thus, high HIF activity drives the lethal cancer phenotype. To effectively treat breast cancer one must eliminate BCSCs and inhibition of HIF-1 activity may aid in achieving this goal. Recently, the HIF-2-selective inhibitor Belzutifan has been approved for the treatment of renal cell carcinoma in patients with von Hippel-Lindau syndrome,^74^ but the studies summarized here suggest this drug will not be effective in blocking hypoxia-induced BCSC specification, which appears to be controlled exclusively by HIF-1. Thus, the development of dual HIF-1/2 inhibitors may provide a more effective therapeutic strategy for eradicating BCSCs.

## Data Availability

No new data were generated or analyzed in support of this review.
